# Assessing the Effectiveness of Digital Health Behavior Strategies on Type 2 Diabetes Management: Systematic Review and Network Meta-Analysis

**DOI:** 10.2196/63209

**Published:** 2025-02-14

**Authors:** Min Li, Shiyu Liu, Binyang Yu, Ning Li, Aili Lyu, Haiyan Yang, Haiyan He, Na Zhang, Jingru Ma, Meichen Sun, Hong Du, Rui Gao

**Affiliations:** 1 School of Nursing Health Science Center Xi 'an Jiaotong University Xi 'an China; 2 School of Public Health Xi’an Jiaotong University Xi 'an China; 3 Graduate School Beijing University of Chinese Medicine Beijing China

**Keywords:** T2DM, type 2 diabetes mellitus, digital health interventions, behavior strategy, strategy combinations, effectiveness, network meta-analysis

## Abstract

**Background:**

Various mobile technologies and digital health interventions (DHIs) have been developed for type 2 diabetes mellitus (T2DM) management. Strategies are crucial for ensuring the effectiveness of DHIs. However, there is currently a lack of categorization and summarization of the strategies used in DHIs for T2DM.

**Objective:**

This study aims to (1) identify and categorize the strategies used in DHIs for T2DM management; (2) assess the effectiveness of these DHI strategies; and (3) compare and rank the efficacy of different strategy combinations on glycated hemoglobin A_1c_ (HbA_1c_) levels, fasting blood glucose (FBG) levels, BMI, and weight loss.

**Methods:**

Relevant randomized controlled trials (RCTs) were extracted from PubMed, Web of Science, and Scopus databases. Three rounds of screening and selection were conducted. The strategies were identified and categorized based on the principles of behavior change techniques and behavior strategies. The synthesis framework for the assessment of health IT was used to structure the evaluation of the DHI strategies qualitatively. A network meta-analysis was performed to compare the efficacy of different strategy combinations. The data quality was assessed using the Cochrane Risk of Bias tool.

**Results:**

A total of 52 RCTs were included, identifying 63 strategies categorized into 19 strategy themes. The most commonly used strategies were *guide*, *monitor*, *management*, and *engagement*. Most studies reported positive or mixed outcomes for most indicators based on the synthesis framework for the assessment of health IT. Research involving a medium or high number of strategies was found to be more effective than research involving a low number of strategies. Of 52 RCTs, 27 (52%) were included in the network meta-analysis. The strategy combination of *communication, engagement, guide*, and *management* was most effective in reducing HbA_1c_ levels (mean difference [MD] –1.04, 95% CI –1.55 to –0.54), while the strategy combination of *guide, management,* and *monitor* was effective in reducing FBG levels (MD –0.96, 95% CI –1.86 to –0.06). The strategy combination of *communication, engagement*, *goal setting, management*, and *support* was most effective for BMI (MD –2.30, 95% CI –3.16 to –1.44) and weight management (MD –6.50, 95% CI –8.82 to –4.18).

**Conclusions:**

Several DHI strategy combinations were effective in reducing HbA_1c_ levels, FBG levels, BMI, and weight in T2DM management. Health care professionals should be encouraged to apply these promising strategy combinations in DHIs during clinical care. Future research should further explore and optimize the design and implementation of strategies.

**Trial Registration:**

PROSPERO CRD42024544629; https://tinyurl.com/3zp2znxt

## Introduction

### The Severity of Type 2 Diabetes

Type 2 diabetes mellitus (T2DM) has become a serious public health problem worldwide. It is a progressive disease that can impair health-related quality of life [1,2] while also imposing substantial economic burdens on individuals, health systems, and society [3]. By 2040, there will be 642 million patients with diabetes worldwide, with the incidence of T2DM on the rise across all regions [4]. T2DM accounts for >90% of the diagnosis in all types of diabetes, and it can cause various complications [5]. The goal of diabetes treatment is to prevent or delay complications and optimize quality of life [6]. Poor glucose control is associated with the occurrence of complications [7]. Glycated hemoglobin A_1c_ (HbA_1c_) can be used to determine glucose control levels [8]. Continuous monitoring of fasting blood glucose (FBG) can provide an intuitive reflection of changes in the patient’s glucose levels. Obesity is closely related to T2DM, and weight gain is an independent risk factor for T2DM [9]. Controlling glucose levels and body weight within the normal range can effectively reduce the complications of patients with diabetes [10]. Many studies have shown that T2DM can be slowed down, stopped, or even reversed by changing lifestyle (eg, low-calorie diet and increasing physical activity) [11,12]. This can reduce long-term complications and may extend life expectancy [13].

### The Effect of Digital Health Interventions

Maintaining glycemic control is a challenge for both patients and health care providers, making it difficult to encourage or motivate patients to make long-term lifestyle changes, explain their self-monitoring of blood glucose data, provide immediate feedback, and understand their lifestyle [14]. Recognizing that patients require more self-management support, various mobile technologies (ie, mobile health care) and digital health interventions (DHIs) have been developed [15-18], including mobile apps, SMS, wearable and ambient sensors, and social media. These technologies can provide early support for the improvement of health behaviors in patients with diabetes, encouraging patients with T2DM to eat healthily, engage in physical exercise, and collect and analyze personal data to assess clinical conditions. The data reported by patients can be used to customize personalized feedback information, including health promotion, motivation, encouragement, reminders, and emotional support information [19,20]. Relevant systematic reviews and meta-analyses have confirmed the effectiveness of DHIs on behavior change, blood glucose control, and weight loss in patients with diabetes [21-24].

### The Critical Role of Strategies in DHIs for T2DM Management

Strategies play a pivotal role in enhancing the efficacy of DHIs for patients with T2DM, with the careful selection and implementation of appropriate strategies being crucial to DHI success [25]. Within the realm of digital interventions, using suitable strategies, such as user-centered participatory design, can heighten user engagement, thereby rendering intervention measures more appealing and efficacious [26]. Furthermore, mobile technology interventions informed by strategies, such as health behavior theory, hold the potential for more comprehensive mechanisms of behavior change, fostering a health care approach that is both impactful and sustainable [27]. Through the identification and categorization of diverse strategies, researchers and practitioners can gain deeper insights into the effective methodologies and techniques used in DHIs. This involves assessing the strengths and limitations of various strategies and their applicability to specific health domains or populations [28].

### Current Findings on DHI Strategies for T2DM Management

There have been studies that systematically summarize the strategies used in DHIs currently. For example, various engagement strategies have been reported in DHIs for mental health promotion, including personalization, human and social support, gamification, personalized feedback, and reminders, which work best to promote engagement [29]. A study indicated that the effectiveness of internet-based interventions correlates with the extensive use of theory, particularly the theory of planned behavior; the incorporation of a greater number of behavior change techniques; and the integration of additional methods for interacting with participants [25]. A systematic review and meta-analysis of lifestyle interventions in postpartum women found that the provision of certain strategies including problem-solving, goal setting of the outcome, reviewing the outcome goal, providing feedback, and self-monitoring of behavior was associated with greater decreases in energy intake [30]. In addition, the review analyzed loneliness reduction strategies including improving social skills, enhancing social support, increasing opportunities for social interaction, and addressing deficits in social cognition [31]. It was suggested in a narrative umbrella review that credible sources, social support, prompts and cues, graded tasks, goals and planning, feedback and monitoring, and human coaching and personalization components increased the effectiveness of DHIs targeting the prevention and management of noncommunicable diseases [32]. Moreover, substantial strategies have been found to be effective in improving recruitment, reducing loss to follow-up, and enhancing retention during intervention in some trials [33-36]. However, there is a lack of categorization and summarization of the strategies used in DHIs of T2DM at present. Although previous research has compared the effectiveness of 5 strategies for T2DM management, it has primarily focused on telemedicine within DHIs and compared several independent strategies, without providing a systematic summary of the strategies used in DHIs [37].” Meanwhile, most interventional studies incorporate different numbers and combinations of strategies rather than focusing on individual strategies, and it is not yet known which strategy combination has the best effect. Therefore, it is essential to summarize and compare strategies and strategy combinations, identify the content of these strategies, and further determine the optimal number and form of strategy combinations to provide more practical guidance for precise and personalized digital health management of T2DM.

### Study Objectives

This study aims to (1) identify and categorize the strategies used in DHIs on T2DM management; (2) assess the effectiveness of these DHI strategies; and (3) compare and rank the efficacy of different strategy combinations on HbA_1c_ levels, FBG levels, BMI, and weight loss.

## Methods

### Protocol and Registration

This systematic review followed the PRISMA-NMA (Preferred Reporting Items for Systematic Review and Meta-Analyses extension for Network Meta-Analysis) 2020 statement for designing and reporting [38]. The PRISMA checklist for this study can be found in Multimedia Appendix 1. The protocol for this study has been registered with PROSPERO (CRD42024544629).

### Data Sources

We conducted a comprehensive search for papers published in English from PubMed, Web of Science, and Scopus databases. This search used a combination of DHI-related terms, along with database-specific subject headings and filters, to ensure thoroughness and focus. The detailed search strategy is provided in Multimedia Appendix 2. The time span was from January 1, 1999, to March 10, 2024.

### Data Selection and Extraction

All the searched records were imported into EndNote X9 (Clarivate) to eliminate duplicate studies. The first round of screening and selection focused on identifying DHIs. Our criteria were as follows:

1. The means of intervention should be digital, primarily including wearable devices, telemedicine, electronic health records, electronic medical records, mobile phone apps, web pages, blogs, emails, SMS text messages, social media, and similar technologies.
2. The intervention must be health related, encompassing areas such as health behavior improvement, disease treatment, and health education.
3. We selected studies that used randomized controlled trials (RCTs) as the intervention as eligible, including both individual and cluster RCTs, as they provide the highest level of evidence for evaluating interventions.

The second round of screening and selection concentrated on identifying specific strategies within the selected DHIs. A strategy is defined as a specific approach or technique used within the intervention to promote health behavior change or improve health outcomes. We used the framework of potential strategies (Multimedia Appendix 3) based on behavior change techniques [39] and behavior strategies [30].

The third round of screening and selection focused on identifying the population with T2DM based on the second round of work. A data extraction form was developed to facilitate electronic comparison of entries. The extracted data include the author, year of publication, study setting (ie, country), characteristics of the participants, details of the interventions, strategies, and outcomes. The inclusion criteria were as follows:

1. Population—patients diagnosed with T2DM and aged ≥18 years
2. Comparisons—the effectiveness of DHIs was compared to no intervention
3. Outcomes—the outcomes for the overview of the effectiveness of DHI strategies included, but were not limited to, acceptability, usability, satisfaction, appropriateness, and efficiency. The outcome of the meta-analysis included the changes in HbA_1c_ (%) level, FBG (mmol/L) level, BMI (kg/m^2^), and weight loss (kg).
4. Study design—we only included RCTs.

During the second and third screening, full manuscripts of the studies identified as potentially relevant were obtained and assessed by 6 independent reviewers according to the inclusion criteria. Any discrepancies were resolved by discussion or through adjudication by a senior researcher.

### Quality Appraisal

Two independent reviewers assessed the individual quality of the final selected studies using the Cochrane Collaboration Risk of Bias tool [40], with any discrepancies being resolved by consensus. The quality evaluation items of each trial included selection bias (random sequence generation and allocation concealment), performance bias (blinding of participants and personnel), detection bias (blinding of outcome assessment), attrition bias (incomplete outcome data), reporting bias (selective reporting), and other bias. Each item was scored as low, high, or unclear risk of bias.

### Data Analysis and Synthesis

#### Methods of Analysis

The variability in some outcomes, such as changes in physical activity, diabetes self-efficacy, medication adherence, quality of life, acceptability, and satisfaction, precluded the possibility of conducting a meta-analysis for these specific measures. Therefore, we reported a structured analysis of the findings to draw conclusions about the effectiveness of different DHI strategies on T2DM management. If a certain outcome measure had a statistically significant (*P*<.05) improvement compared to the control group or over time, it was considered effective. If the outcome measures did not show significant changes over time or there was no statistically significant difference from the control group, it was considered that there was not enough evidence to prove their effectiveness. The synthesis framework for the assessment of health IT (SF/HIT) was used to structure the evaluation of the studies because it included a whole system set of outcome variables [41]. These comprised variables such as adherence or attendance, acceptability, effectiveness, satisfaction, and perceived ease of use or usefulness. Following the framework, evidence for each of the outcome variables was coded as *positive or mixed* or *neutral or negative.* If the study did not address the outcome in question, it was coded as neutral or negative.

For the meta-analysis, the outcome included HbA_1c_ levels, FBG levels, BMI, and weight loss. These were continuous variables, and thus, network estimates were presented as mean differences (MDs) with 95% CIs. We assumed that a *P* value<.05 indicated statistical significance. We measured the level of heterogeneity with the *I*^2^ statistics; an *I*^2^<50% was considered to have no significant heterogeneity, in which case we would use a fixed-effects model to calculate the pooled effect sizes. Otherwise, a random-effects model would be used. Stata software (version 15.1, *network package* and *network graphs package*; StataCorp) was used to conduct network meta-analysis [42,43]. The *network package* performed the network meta-analysis based on the frequentist framework using random-effects models. The approach was to test the research hypothesis, as this was simpler than the problem of establishing prior probability [44]. This approach is not complex and has few limitations for ordinary researchers using network meta-analysis [45]. A network diagram with nodes and lines was constructed to represent different interventions, where the size of nodes represents the number of populations, and the thickness of lines between nodes represents the number of studies. The results of the network meta-analysis were summarized based on all possible pairwise comparisons, including mixed comparisons (ie, the combined effect of direct and indirect comparisons) and indirect comparisons. The effect of different interventions was estimated based on the surface under the cumulative ranking curve (SUCRA). The SUCRA value ranges from 0% to 100%, where a SUCRA value of 100% indicates that the treatment was the most effective, and the smaller the value, the poorer the treatment effect.

#### Assessment of Inconsistency

The node-splitting test was used to assess the local inconsistency between direct and indirect comparisons. Differences between direct and indirect coefficients (assessed via the *P* value) were used to estimate inconsistency: if *P*<.05, local inconsistency was considered to exist [46]. If inconsistency was observed, nontransitivity was also suspected to exist, and potential modifiers influencing treatment effects were examined.

#### Risk of Bias Across Studies

The risk of publication bias in network meta-analysis was analyzed using the Egger test [47]. The symmetry of the generated funnel plots was assessed visually using the Egger test, together with adjusted rank correlation and regression asymmetry tests [48,49].

#### Sensitivity Analyses

We used the method of removing individual studies separately.

## Results

### Characteristics of Included Studies

**Figure 1:**
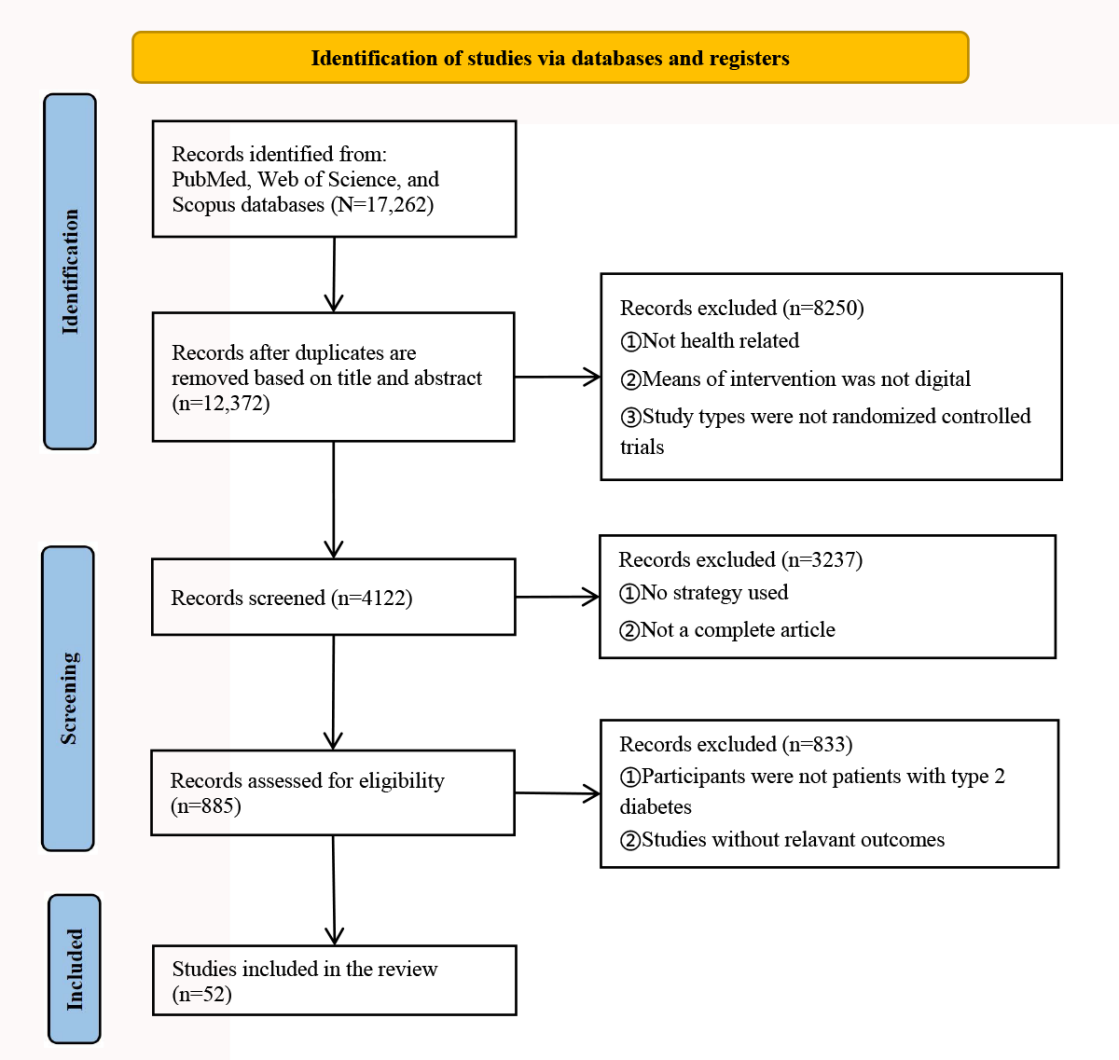
Flow diagram for the search and selection of the included studies.

The schematic flow for the selection of the included studies is presented in Figure 1. A total of 12,372 studies were identified, of which only 52 (0.42%) trials [14,50-100] met the eligibility criteria and were included. The characteristics of the included studies are provided in Multimedia Appendix 4 [14,50-100]. Among the 52 included studies from the literature, the publication year was between 2000 and 2023. The number of publications has also increased over the years, with the highest publication volume in 2022 and 2023. Most research was conducted in the United States (20/52, 38%), followed by China (5/52, 10%). The minimum sample size was 30, and the maximum was 1926. The duration of intervention ranged from 3 to 24 months, with most studies lasting 12 months (15/52, 29%). The outcome indicators in intervention mainly included HbA_1c_ levels, FBG levels, BMI, blood pressure, weight loss, waist circumference, low-density lipoprotein cholesterol levels, high-density lipoprotein cholesterol levels, total cholesterol levels, diabetes self-efficiency, diabetes medication adherence, quality of life, self-management, depression, diabetes distress, and other outcomes.

### Synthesis of Different DHI Strategies

Among the 52 studies included, we identified 63 different strategies, and they were categorized into 19 themes. Among the identified strategies, *monitor* and *guide* were the most frequently used, appearing in 77% (40/52) and 67% (35/52) of the total papers, respectively. Following closely was *management* (32/52, 61%) and *engagement* (28/52, 54%). More than 40% of the studies used *stimulate,* *communication,* and *goal setting.* In addition, more than one-fifth of the research used *support,* *shape, feedback, prompt, action,* and *tailor*. The usage frequencies were notably lower for cues, *identity*, *reward*, *model or demonstrate*, and *restructure.* The studies ranged from using a minimum of 1 to a maximum of 32 strategies. We categorized studies into 3 groups based on the number of strategies used: low (1 to 3 strategies), medium (4 to 6 strategies), and high (≥7 strategies). Among the 52 studies, 18 (35%) were categorized as high-strategy study, 23 (44%) as medium-strategy study, and 11 (21%) as low-strategy study. In these 52 studies, 37 different combinations of strategies were identified based on the thematic strategies used in each study. Detailed information about these strategies and strategy combinations is provided in Multimedia Appendix 5. The strategy themes and the number of identified studies are listed in Table 1.

**Table 1:** The strategy themes and the number of identified studies (N=52).

	Theme	Studies, n (%)
A	Action planning	12 (23)
B	Communication	23 (44)
C	Cues	4 (8)
D	Engagement	28 (54)
E	Feedback	13 (25)
F	Goal setting	24 (46)
G	Guide	35 (67)
H	Identity	4 (8)
I	Management	32 (61)
J	Model or demonstrate	2 (4)
K	Monitor	40 (77)
L	Prompt	12 (23)
M	Restructure	2 (4)
N	Reward	4 (8)
O	Shape	15 (29)
P	Stimulate	22 (42)
Q	Support	19 (36)
R	Tailor	11 (21)
S	Others	7 (13)

### Evaluation of the Effectiveness of Different DHI Strategies

In this assessment, we encoded positive or mixed as 1 and neutral or negative as 0. Overall, most studies have reported positive or mixed outcomes for most outcome indicators. The number of positive or mixed results that studies achieved ranged from 6 to 11. In total, 41 (79%) of the 52 studies have reported having ≥10 positive or mixed results as stipulated in the SF/HIT. For the preventive care; efficiency; perceived ease of use or usefulness; safety, privacy, or security; acceptability; appropriateness; and satisfaction domains nearly all studies (>50) have reported positive or mixed results. Following closely were the process of service delivery or performance, effectiveness, and adherence or attendance domains, with 49 (94%), 47 (90%), and 45 (86%) of the 52 studies reporting positive or mixed results. However, in terms of cost-effectiveness, most studies (35/52, 67%) either did not report relevant results or reported neutral or negative findings. Out of 18 high-strategy studies, 16 (89%) reported ≥10 positive or mixed results. Out of 23 medium-strategy studies, 20 (87%) reported ≥10 positive or mixed results. However, 7 (64%) out of the 11 low-strategy studies reported having ≥10 positive or mixed results. Meanwhile, it is noteworthy that the 2 studies with the fewest positive or mixed results were both high-strategy studies. The evaluation summary of SF/HIT results is presented in Multimedia Appendix 6.

### Risk of Bias and Quality Assessments of Included Studies

**Figure 2:**
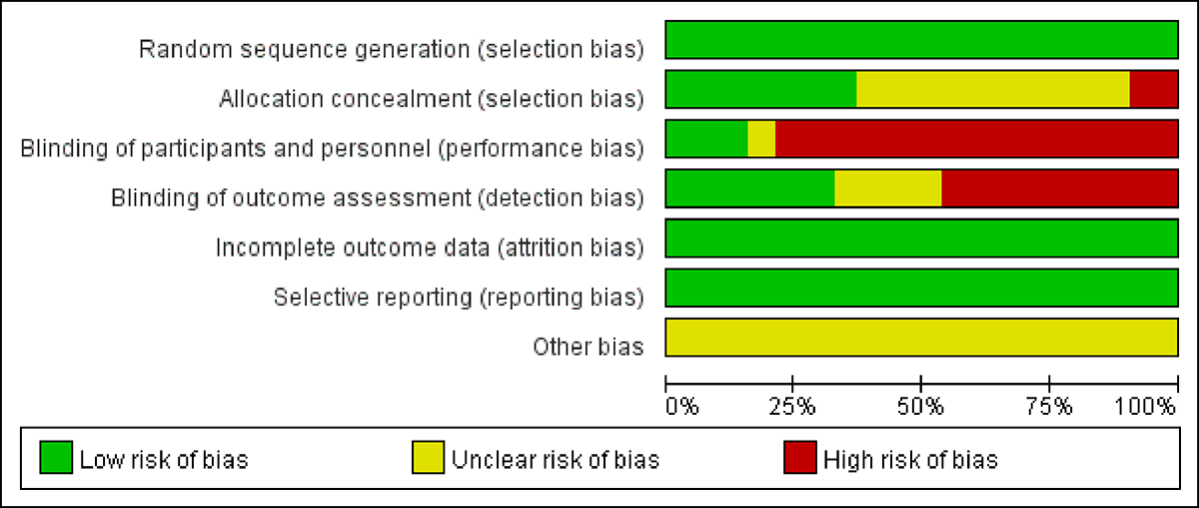
Risk of bias graph: review authors’ judgments about each risk of bias item presented as percentages across all included studies.

We conducted a synthesis of bias risk assessment results using RevMan software (version 5.4; Cochrane), as shown in Figure 2. Overall, all included RCTs exhibited a relatively low risk of bias. All studies reported the generation of random sequences and described specific randomization methods assessed as low risk. There were no incomplete outcome data across all studies assessed as low risk. Selective reporting was not observed in any of the studies assessed as low risk. Regarding allocation concealment, 36% (19/52) of the studies reported using this method, assessed as low risk; 54% (28/52) did not provide such reporting, assessed as unclear risk; and 10% (5/52) explicitly stated not using allocation concealment, assessed as high risk. For blinding of participants and personnel, most studies (41/52, 79%) did not use blinding, assessed as high risk, often due to the nature of the intervention precluding blinding; 15% (8/52) of the studies implemented blinding for participants, assessed as low risk, while 6% (3/52) did not report blinding status, assessed as unclear risk. For the blinding of outcome assessment, 46% (24/52) of the studies did not use blinding, resulting in high risk, whereas 33% (17/52) reported using blinding for outcome assessment, assessed as low risk, with the remainder (11/52, 21%) assessed as unclear risk. The risk of bias in each study is presented in Multimedia Appendix 7.

### Meta-Analysis

#### Effects of Strategy Combinations in Reducing HbA1c Levels

Of 52 included RCTs, 27 (52%) RCTs [14,50,53-55,57,59,60,66,68,70,72,74,77,78,80,82,84,85,87,88,93,94,96-98,100] assessed the effects of 12 different DHI strategy combinations on HbA_1c_ levels in patients with T2DM. These 12 strategy combinations encompassed (1) ABCDEFGHIJKLMNOQR (action planning, communication, cues, engagement, feedback, goal setting, guide, identity, management, model or demonstrate, monitor, prompt, restructure, reward, shape, support, and tailor); (2) ABDEFGIKLOPQ (action planning, communication, engagement, feedback, goal setting, guide, management, monitor, prompt, shape, stimulate, and support); (3) FGIKQ (goal setting, guide, management, monitor, and support); (4) BGKR (communication, guide, monitor, and tailor); (5) EGIKPR (feedback, guide, management, monitor, stimulate, and tailor); (6) EKP (feedback, monitor, and stimulate); (7) DGIKPQR (engagement, guide, management, monitor, stimulate, support, and tailor); (8) BDFIQ (communication, engagement, goal setting, management, and support); (9) BDGI (communication, engagement, guide, and management); (10) BDGKNOP (communication, engagement, guide, monitor, reward, shape, and stimulate); (11) DFGKP (engagement, goal setting, guide, monitor, and stimulate); and (12) GIK (guide, management, and monitor). The specific strategy represented by the letters in each strategy combination is provided in Table 1.

**Figure 3:**
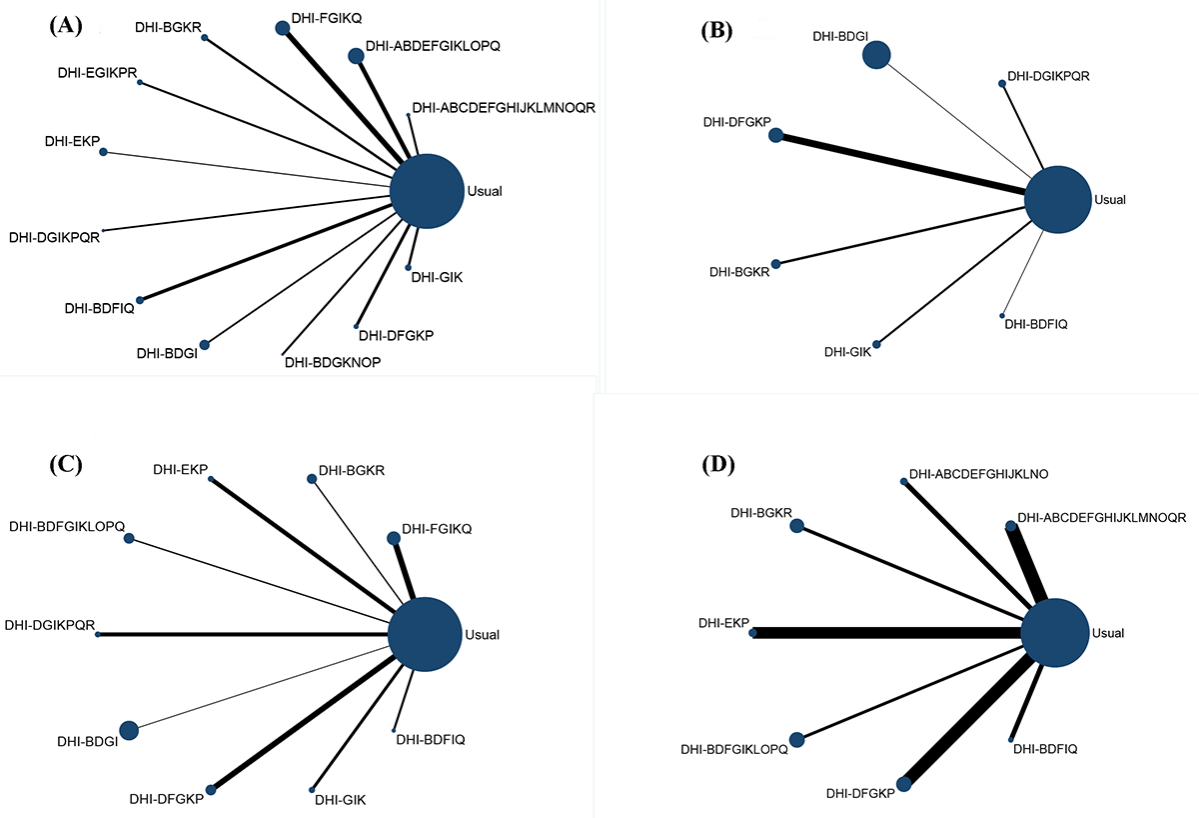
Evidence network of outcome indicators: (A) evidence network for glycated hemoglobin A1c, (B) evidence network for fasting blood glucose, (C) evidence network for BMI, and (4) evidence network for weight loss. ABDEFGIKLOPQ: action planning, communication, engagement, feedback, goal setting, guide, management, monitor, prompt, shape, stimulate, and support; ABCDEFGHIJKLNO: action planning, communication, cues, engagement, feedback, goal setting, guide, identity, management, model or demonstrate, monitor, prompt, reward, and shape; ABCDEFGHIJKLMNOQR: action planning, communication, cues, engagement, feedback, goal setting, guide, identity, management, model or demonstrate, monitor, prompt, restructure, reward, shape, support, and tailor; BDFGIKLOPQ: communication, engagement, goal setting, guide, management, monitor, prompt, shape, stimulate, and support; BDFIQ: communication, engagement, goal setting, management, and support; BDGI: communication, engagement, guide, and management; BDGKNOP: communication, engagement, guide, monitor, reward, shape, and stimulate; BGKR: communication, guide, monitor, and tailor; DFGKP: engagement, goal setting, guide, monitor, and stimulate; DGIKPQR: engagement, guide, management, monitor, stimulate, support, and tailor; DHI: digital health intervention; EGIKPR: feedback, guide, management, monitor, stimulate, and tailor; EKP: feedback, monitor, and stimulate; FGIKQ: goal setting, guide, management, monitor, and support; GIK: guide, management, and monitor.

**Figure 4:**
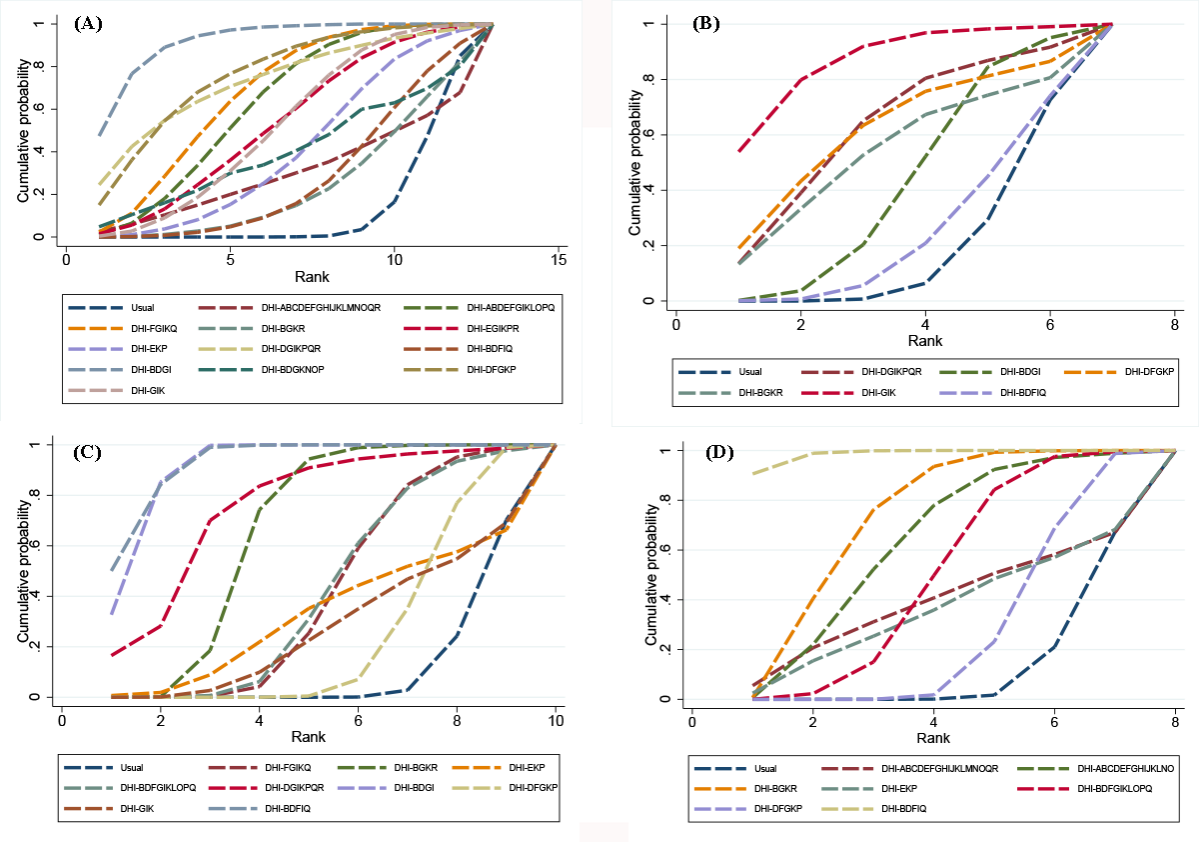
Surface under the cumulative ranking curve rank of outcome indicators: (A) surface under the cumulative ranking curve rank for glycated hemoglobin A1c, (B) surface under the cumulative ranking curve rank for fasting blood glucose, (C) surface under the cumulative ranking curve rank for BMI, and (D) surface under the cumulative ranking curve rank for weight loss. ABDEFGIKLOPQ: action planning, communication, engagement, feedback, goal setting, guide, management, monitor, prompt, shape, stimulate, and support; ABCDEFGHIJKLNO: action planning, communication, cues, engagement, feedback, goal setting, guide, identity, management, model or demonstrate, monitor, prompt, reward, and shape; ABCDEFGHIJKLMNOQR: action planning, communication, cues, engagement, feedback, goal setting, guide, identity, management, model or demonstrate, monitor, prompt, restructure, reward, shape, support, and tailor; BDFGIKLOPQ: communication, engagement, goal setting, guide, management, monitor, prompt, shape, stimulate, and support; BDFIQ: communication, engagement, goal setting, management, and support; BDGI: communication, engagement, guide, and management; BDGKNOP: communication, engagement, guide, monitor, reward, shape, and stimulate; BGKR: communication, guide, monitor, and tailor; DFGKP: engagement, goal setting, guide, monitor, and stimulate; DGIKPQR: engagement, guide, management, monitor, stimulate, support, and tailor; DHI: digital health intervention; EGIKPR: feedback, guide, management, monitor, stimulate, and tailor; EKP: feedback, monitor, and stimulate; FGIKQ: goal setting, guide, management, monitor, and support; GIK: guide, management, and monitor.

**Figure 5:**
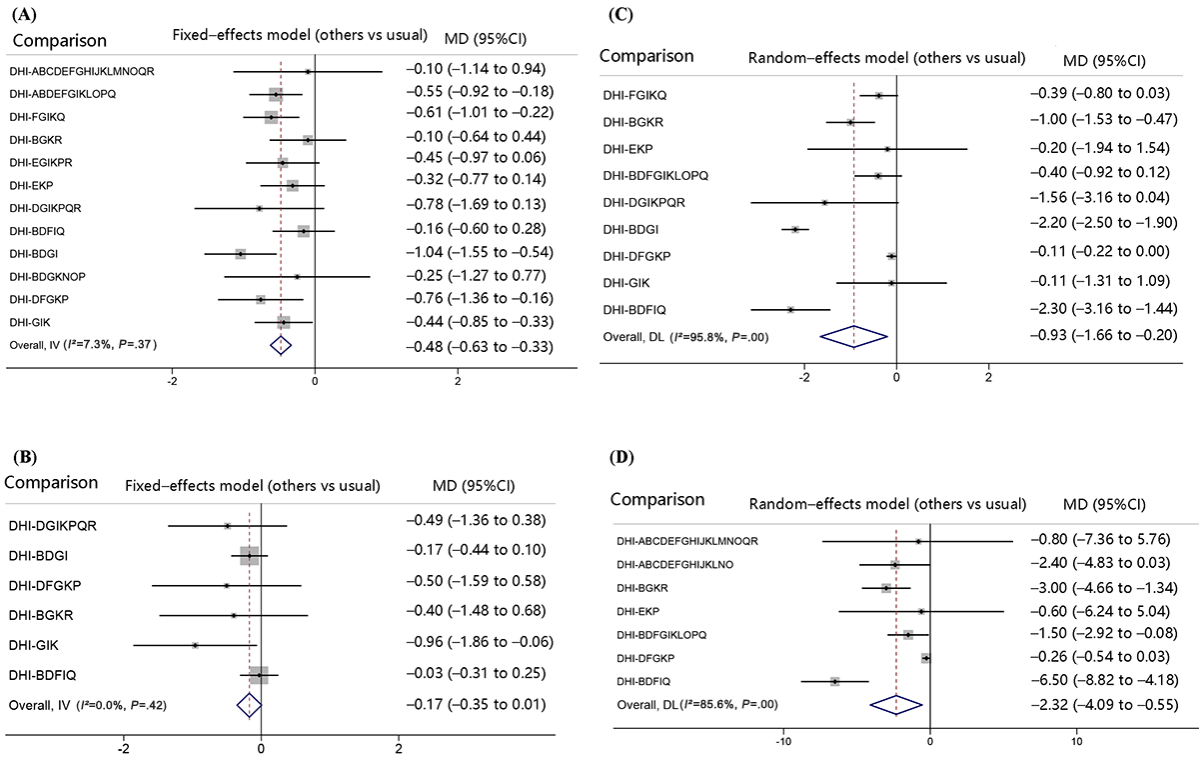
Forest plots of network meta-analysis results: (A) forest plots for glycated hemoglobin A1c, (B) forest plots for fasting blood glucose; (C) forest plots for BMI, and (D) forest plots for weight loss. ABDEFGIKLOPQ: action planning, communication, engagement, feedback, goal setting, guide, management, monitor, prompt, shape, stimulate, and support; ABCDEFGHIJKLNO: action planning, communication, cues, engagement, feedback, goal setting, guide, identity, management, model or demonstrate, monitor, prompt, reward, and shape; ABCDEFGHIJKLMNOQR: action planning, communication, cues, engagement, feedback, goal setting, guide, identity, management, model or demonstrate, monitor, prompt, restructure, reward, shape, support, and tailor; BDFGIKLOPQ: communication, engagement, goal setting, guide, management, monitor, prompt, shape, stimulate, and support; BDFIQ: communication, engagement, goal setting, management, and support; BDGI: communication, engagement, guide, and management; BDGKNOP: communication, engagement, guide, monitor, reward, shape, and stimulate; BGKR: communication, guide, monitor, and tailor; DFGKP: engagement, goal setting, guide, monitor, and stimulate; DGIKPQR: engagement, guide, management, monitor, stimulate, support, and tailor; DHI: digital health intervention; DL: DerSimonian and Laird method; EGIKPR: feedback, guide, management, monitor, stimulate, and tailor; EKP: feedback, monitor, and stimulate; FGIKQ: goal setting, guide, management, monitor, and support; GIK: guide, management, and monitor; IV: inverse variance method; MD: mean difference.

The network evidence plot for HbA_1c_ levels is shown in Figure 3A. The SUCRA probability ranking for the reducing effect of HbA_1c_ levels in different DHI strategy combinations is shown in Figure 4A. Of the 12 DHI strategy combinations, the possibility of DHI-BDGI (communication, engagement, guide, and management) being the best strategy combination was the highest. The SUCRA value predicted the possibility of different strategy combinations as the best way, and the effects were ranked as follows: DHI-BDGI (91.8%)>DHI-DFGKP (76%)>DHI-DGIKPQR (73.2%)>DHI-FGIKQ (67.4%)>DHI-ABDEFGIKLOPQ (62.1%) > DHI-EGIKPR (52.8%)>DHI-GIK (52.1%)>DHI-EKP (40.5%)>DHI-BDGKNOP (39.4%)>DHI-ABCDEFGHIJKLMNOQR (30.1%)>DHI-BDFIQ (27.7%)>DHI-BGKR (24.1%)>usual (12.8%). Compared with the usual care groups, DHI-BDGI (MD –1.04, 95% CI –1.55 to –0.54), DHI-DFGKP (MD –0.76, 95% CI –1.36 to –0.16), DHI-FGIKQ (MD –0.61, 95% CI –1.01 to –0.22), DHI-ABDEFGIKLOPQ (MD –0.55, 95% CI –0.92 to –0.18), and DHI-GIK (MD –0.44, 95% CI –0.85 to –0.03) strategy combinations were statistically significantly effective in reducing HbA_1c_ levels (Figure 5A). On the basis of the interval estimation of direct and indirect comparison (Table 2), DHI-BDGI was more effective than the DHI-EKP, DHI-BDFIQ, and DHI-BGKR strategy combinations.

**Table 2:** Network meta-analysis of glycated hemoglobin A1c levels.

DHI^a^ strategy combinations	MDs^b^ (95% CIs)												
	DHI-BDGI^c^	DHI-DFGKP^d^	DHI-DGIKPQR^e^	DHI-FGIKQ^f^	DHI-ABDEFGIKLOPQ^g^	DHI-EGIKPR^h^	DHI-GIK^i^	DHI-EKP^j^	DHI-BDGKNOP^k^	DHI**-**ABCDEFGHIJKLMNOQR^l^	DHI-BDFIQ^m^	DHI-BGKR^n^	Usual
DHI-DFGKP	–0.28 (–1.06 to 0.50)	0	—^o^	—	—	—	—	—	—	—	—	—	—
DHI-DGIKPQR	–0.26 (–1.30 to 0.78)	0.02 (–1.07 to 1.11)	0	—	—	—	—	—	—	—	—	—	—
DHI-FGIKQ	–0.43 (–1.07 to 0.21)	–0.15 (–0.86 to 0.57)	–0.17 (–1.16 to 0.82)	0	—	—	—	—	—	—	—	—	—
DHI-ABDEFGIKLOPQ	–0.50 (–1.12 to 0.13)	–0.21 (–0.92 to 0.49)	–0.23 (–1.21 to 0.75)	–0.07 (–0.61 to 0.48)	0	—	—	—	—	—	—	—	—
DHI-EGIKPR	–0.59 (–1.31 to 0.13)	–0.31 (–1.10 to 0.48)	–0.33 (–1.37 to 0.72)	–0.16 (–0.81 to 0.49)	–0.10 (–0.73 to 0.54)	0	—	—	—	—	—	—	—
DHI-GIK	–0.60 (–1.24 to 0.03)	–0.32 (–1.05 to 0.40)	–0.34 (–1.34 to 0.65)	–0.17 (–0.74 to 0.39)	–0.11 (–0.66 to 0.44)	–0.01 (–0.67 to 0.64)	0	—	—	—	—	—	—
DHI-EKP	–0.73 (–1.41 to –0.05)	–0.45 (–1.19 to 0.30)	–0.46 (–1.48 to 0.55)	–0.30 (–0.90 to 0.30)	–0.23 (–0.82 to 0.35)	–0.14 (–0.82 to 0.55)	–0.12 (–0.73 to 0.48)	0	—	—	—	—	—
DHI-BDGKNOP	–0.79 (–1.93 to 0.35)	–0.51 (–1.69 to 0.67)	–0.53 (–1.90 to 0.84)	–0.36 (–1.46 to 0.73)	–0.30 (–1.38 to 0.79)	–0.20 (–1.35 to 0.94)	–0.19 (–1.29 to 0.91)	–0.07 (–1.18 to 1.05)	0	—	—	—	—
DHI**-**ABCDEFGHIJKLMNOQR	–0.94 (–2.10 to 0.22)	–0.66 (–1.86 to 0.54)	–0.68 (–2.06 to 0.70)	–0.51 (–1.63 to 0.60)	–0.45 (–1.56 to 0.66)	–0.35 (–1.52 to 0.81)	–0.34 (–1.46 to 0.78)	–0.22 (–1.35 to 0.92)	–0.15 (–1.61 to 1.31)	0	—	—	—
DHI-BDFIQ	–0.88 (–1.55 to –0.22)	–0.60 (–1.34 to 0.14)	–0.62 (–1.63 to 0.39)	–0.45 (–1.04 to 0.13)	–0.39 (–0.96 to 0.18)	–0.29 (-0.97 to 0.38)	–0.28 (–0.87 to 0.31)	–0.16 (–0.78 to 0.47)	–0.09 (–1.20 to 1.02)	0.06 (–1.07 to 1.19)	0	—	—
DHI-BGKR	–0.94 (–1.68 to –0.21)	–0.66 (–1.46 to 0.14)	–0.68 (–1.73 to 0.37)	–0.51 (–1.18 to 0.15)	–0.45 (–1.10 to 0.20)	−0.35 (–1.10 to 0.39)	–0.34 (–1.01 to 0.33)	–0.22 (–0.92 to 0.49)	–0.15 (–1.30 to 1.00)	0.00 (–1.17 to 1.17)	–0.06 (–0.75 to 0.63)	0	—
Usual	–1.04 (–1.55 to –0.54)	–0.76 (–1.36 to –0.16)	–0.78 (–1.69 to 0.13)	–0.61 (–−1.01 to –0.22)	–0.55 (–0.92 to –0.18)	–0.45 (–0.97 to 0.06)	–0.44 (–0.85 to –0.03)	–0.32 (–0.77 to 0.14)	–0.25 (–1.27 to 0.77)	–0.10 (–1.14 to 0.94)	–0.16 (–0.60 to 0.28)	–0.10 (–0.64 t 0.44)	0

^a^DHI: digital health intervention.

^b^MD: mean difference.

^c^BDGI: communication, engagement, guide, and management.

^d^DFGKP: engagement, goal setting, guide, monitor, and stimulate.

^e^DGIKPQR: engagement, guide, management, monitor, stimulate, support, and tailor.

^f^FGIKQ: goal setting, guide, management, monitor, and support.

^g^ABDEFGIKLOPQ: action planning, communication, engagement, feedback, goal setting, guide, management, monitor, prompt, shape, stimulate, and support.

^h^EGIKPR: feedback, guide, management, monitor, stimulate, and tailor.

^i^GIK: guide, management, and monitor.

^j^EKP: feedback, monitor, and stimulate.

^k^BDGKNOP: communication, engagement, guide, monitor, reward, shape, and stimulate.

^l^ABCDEFGHIJKLMNOQR: action planning, communication, cues, engagement, feedback, goal setting, guide, identity, management, model or demonstrate, monitor, prompt, restructure, reward, shape, support, and tailor.

^m^BDFIQ: communication, engagement, goal setting, management, and support.

^n^BGKR: communication, guide, monitor, and tailor.

^o^Not applicable.

#### Effects of Strategy Combinations on FBG Levels

Of 52 included RCTs, 7 (13%) RCTs [74,78,84,85,87,97,98] assessed the effects of 6 different DHI strategy combinations on FBG levels in patients with T2DM. These 6 strategy combinations encompassed (1) DGIKPQR (engagement, guide, management, monitor, stimulate, support, and tailor); (2) BDGI (communication, engagement, guide, and management); (3) DFGKP (engagement, goal setting, guide, monitor, and stimulate); (4) BGKR (communication, guide, monitor, and tailor); (5) GIK (guide, management, and monitor); and (6) BDFIQ (communication, engagement, goal setting, management, and support).

The network evidence plot for FBG levels is shown in Figure 3B. The SUCRA probability ranking for the reducing effect of FBG levels of different DHI strategy combinations is shown in Figure 4B. Of the 6 strategy combinations, the possibility of DHI-GIK (guide, management, and monitor) being the best strategy combination was the highest. The SUCRA value predicted the possibility of different strategy combinations as the best way, and the effects were ranked as follows: DHI-GIK (86.7%)>DHI-DGIKPQR (62.8%)>DHI-DFGKP (61.6%)>DHI-BGKR (53.6%)>DHI-BDGI (42.7%)>DHI-BDFIQ (24.4%)>usual (18.2%). Compared with the usual care groups, only the DHI-GIK strategy combination (MD –0.96, 95% CI –1.86 to –0.06) was statistically significantly effective in reducing FBG levels (Figure 5B). On the basis of the interval estimation of direct and indirect comparison (Table 3), no strategy combination was superior to the others.

**Table 3:** Network meta-analysis of fasting blood glucose levels.

DHI^a^ strategy combinations	MDs^b^ (95% CIs)						
	DHI-GIK^c^	DHI-DGIKPQR^d^	DHI-DFGKP^e^	DHI-BGKR^f^	DHI-BDGI^g^	DHI-BDFIQ^h^	Usual
DHI-DGIKPQR	–0.47 (–1.72 to 0.78)	0	—^i^	—	—	—	—
DHI-DFGKP	–0.46 (-1.87 to 0.95)	0.01 (–1.38 to 1.40)	0	—	—	—	—
DHI-BGKR	–0.56 (–1.97 to 0.85)	–0.09 (–1.47 to 1.29)	–0.10 (–1.64 to 1.43)	0	—	—	—
DHI-BDGI	–0.79 (–1.73 to 0.15)	–0.32 (–1.23 to 0.59)	–0.33 (–1.45 to 0.79)	–0.23 (–1.34 to 0.88)	0	—	—
DHI-BDFIQ	–0.93 (–1.87 to 0.01)	–0.46 (–1.37 to 0.45)	–0.47 (–1.60 to 0.65)	–0.37 (–1.49 to 0.75)	–0.14 (–0.53 to 0.25)	0	—
Usual	–0.96 (–1.86 to –0.06)	–0.49 (–1.36 to 0.38)	–0.50 (–1.59 to 0.58)	–0.40 (–1.48 to 0.68)	–0.17 (–0.44 to 0.10)	–0.03 (–0.31 to 0.25)	0

^a^DHI: digital health intervention.

^b^MD: mean difference.

^c^GIK: guide, management, and monitor.

^d^DGIKPQR: engagement, guide, management, monitor, stimulate, support, and tailor.

^e^DFGKP: engagement, goal setting, guide, monitor, and stimulate.

^f^BGKR: communication, guide, monitor, and tailor.

^g^BDGI: communication, engagement, guide, and management.

^h^BDFIQ: communication, engagement, goal setting, management, and support.

^i^Not applicable.

#### Effects of Strategy Combinations on BMI

Of 52 included RCTs, 11 (21%) RCTs [60,66,70,72-74,78,84,85,97,98] assessed the effects of 9 different DHI strategy combinations on BMI levels in patients with T2DM. These 9 strategy combinations encompassed (1) FGIKQ (goal setting, guide, management, monitor, and support); (2) BGKR (communication, guide, monitor, and tailor); (3) EKP (feedback, monitor, and stimulate); (4) BDFGIKLOPQ (communication, engagement, goal setting, guide, management, monitor, prompt, shape, stimulate, and support); (5) DGIKPQR (engagement, guide, management, monitor, stimulate, support, and tailor); (6) BDGI (communication, engagement, guide, and management); (7) DFGKP (engagement, goal setting, guide, monitor, and stimulate); (8) GIK (guide, management, and monitor); and (9) BDFIQ (communication, engagement, goal setting, management, and support).

The network evidence plot for BMI is shown in Figure 3C. The SUCRA probability ranking for the reducing effect of BMI of different DHI strategy combinations is shown in Figure 4C. Of the 9 strategy combinations, DHI-BDFIQ (communication, engagement, goal setting, management, and support) was most likely to be the optimal strategy combination. On the basis of the SUCRA values, the effects were ranked as follows: DHI-BDFIQ (92.6%)>DHI-BDGI (90.9%)>DHI-DGIKPQR (75.2%)>DHI-BGKR (65.1%)>DHI-BDFGIKLOPQ (41.4%)>DHI-FGIKQ (40.9%)>DHI-EKP (32%)>DHI-GIK (26.8%)>DHI-DFGKP (24.3%)>Usual (10.8%). Compared with the usual care groups, DHI-BDFIQ (MD –2.30, 95% CI –3.16 to –1.44), DHI-BDGI (MD –2.20, 95% CI –2.50 to –1.90) and DHI-BGKR (MD –1.00, 95% CI –1.53 to –−0.47) strategy combinations were statistically significantly effective in reducing BMI (Figure 5C). On the basis of the interval estimation of direct and indirect comparison (Table 4), DHI-BDFIQ was superior to all other strategy combinations except for DHI-BDGI and DHI-DGIKPQR. In addition, except for DHI-BDFIQ and DHI-DGIKPQR, DHI-BDGI was superior to the rest of strategy combinations. DHI-BGKR was also superior to the DHI-DFGKP strategy combination.

**Table 4:** Network meta-analysis of BMI.

DHI^a^ strategy combinations	MDs^b^ (95% CIs)									
	DHI-BDFIQ^c^	DHI-BDGI^d^	DHI-DGIKPQR^e^	DHI-BGKR^f^	DHI-BDFGIKLOPQ^g^	DHI-FGIKQ^h^	DHI-EKP^i^	DHI-GIK^j^	DHI-DFGKP^k^	Usual

DHI-BDGI	–0.10 (–1.01 to 0.81)	0	—^l^	—	—	—	—	—	—	—
DHI-DGIKPQR	–0.74 (–2.56 to 1.08)	–0.64 (–2.27 to 0.99)	0	—	—	—	—	—	—	—
DHI-BGKR	–1.30 (–2.31 to –0.29)	–1.20 (–1.81 to –0.59)	–0.56 (–2.25 to 1.13)	0	—	—	—	—	—	—
DHI-BDFGIKLOPQ	–1.90 (–2.90 to –0.90)	–1.80 (–2.40 to –1.20)	–1.16 (–2.85 to 0.53)	–0.60 (–1.34 to 0.14)	0	—	—	—	—	—
DHI-FGIKQ	–1.91 (–2.87 to –0.96)	–1.81 (–2.33 to –1.30)	–1.17 (–2.83 to 0.48)	–0.61 (–1.29 to 0.06)	–0.01 (–0.68 to 0.65)	0	—	—	—	—
DHI-EKP	–2.10 (–4.04 to –0.16)	–2.00 (–3.76 to –0.24)	–1.36 (–3.72 to 1.00)	–0.80 (–2.62 to 1.02)	–0.20 (–2.01 to 1.61)	–0.19 (1.97 to 1.60)	0	—	—	—
DHI-GIK	−2.19 (−3.67 to −0.71)	−2.09 (−3.33 to −0.85)	–1.45 (–3.45 to 0.55)	–0.89 (–2.20 to 0.42)	–0.29 (–1.60 to 1.02)	–0.28 (–1.55 to 0.99)	–0.09 (–2.20 to 2.02)	0	—	—
DHI-DFGKP	−2.19 (−3.06 to −1.32)	−2.09 (−2.41 to −1.77)	–1.45 (–3.06 to 0.16)	–0.89 (–1.43 to –0.35)	–0.29 (–0.82 to 0.24)	–0.28 (–0.71 to 0.15)	–0.09 (–1.83 to 1.65)	–0.00 (–1.21 to 1.20)	0	—
Usual	−2.30 (−3.16 to −1.44)	−2.20 (−2.50 to −1.90)	–1.56 (–3.16 to 0.04)	–1.00 (–1.53 to –0.47)	–0.40 (–0.92 to 0.12)	–0.39 (–0.80 to 0.03)	–0.20 (–1.94 to 1.54)	–0.11 (–1.31 to 1.09)	–0.11 (–0.22 to 0.00)	0

^a^DHI: digital health intervention.

^b^MD: mean difference.

^c^BDFIQ: communication, engagement, goal setting, management, and support.

^d^BDGI: communication, engagement, guide, and management.

^e^DGIKPQR: engagement, guide, management, monitor, stimulate, support, and tailor.

^f^BGKR: communication, guide, monitor, and tailor.

^g^BDFGIKLOPQ: communication, engagement, goal setting, guide, management, monitor, prompt, shape, stimulate, and support.

^h^FGIKQ: goal setting, guide, management, monitor, and support.

^i^EKP: feedback, monitor, and stimulate.

^j^GIK: guide, management, and monitor.

^k^DFGKP: engagement, goal setting, guide, monitor, and stimulate.

^l^Not applicable.

#### Effects of Strategy Combinations on Weight Loss

Of 52 included RCTs, 8 (15%) RCTs [50,52,70,72,73,84,85,98] assessed the effects of 7 different DHI strategy combinations on weight loss in patients with T2DM. These 7 strategy combinations encompassed (1) ABCDEFGHIJKLMNOQR (action planning, communication, cues, engagement, feedback, goal setting, guide, identity, management, model or demonstrate, monitor, prompt, restructure, reward, shape, support, and tailor); (2) ABCDEFGHIJKLNO (action planning, communication, cues, engagement, feedback, goal setting, guide, identity, management, model or demonstrate, monitor, prompt, reward, and shape); (3) BGKR (communication, guide, monitor, and tailor); (4) EKP (feedback, monitor, and stimulate); (5) BDFGIKLOPQ (communication, engagement, goal setting, guide, management, monitor, prompt, shape, stimulate, and support); (6) DFGKP (engagement, goal setting, guide, monitor, and stimulate); (7) BDFIQ (communication, engagement, goal setting, management, and support).

The network evidence plot for weight loss is shown in Figure 3D. The SUCRA probability ranking for the effect of weight loss of different DHI strategy combinations is shown in Figure 4D. Of the 7 strategy combinations, DHI-BDFIQ (communication, engagement, goal setting, management, support) was most likely to be the optimal strategy combination. On the basis of the SUCRA values, the effects were ranked as follows: DHI-BDFIQ (98.5%)>DHI-BGKR (72.9%)>DHI-ABCDEFGHIJKLNO (63%)>DHI-BDFGIKLOPQ (49.8%)>DHI-ABCDEFGHIJKLMNOQR (39.2%)>DHI-EKP (36.2%)>DHI-DFGKP (27.5%)>Usual (12.9%). Compared with the usual care groups, DHI-BDFIQ (MD –6.50, 95% CI –8.82 to –4.18), DHI-BGKR (MD –3.00, 95% CI –4.66 to –1.34) and DHI-BDFGIKLOPQ (MD –1.50, 95% CI –2.92 to –0.08) strategy combinations were statistically significantly effective in weight loss (Figure 5D). On the basis of the interval estimation of direct and indirect comparison (Table 5), DHI-BDFIQ was superior to DHI-BGKR, DHI-ABCDEFGHIJKLNO, DHI-BDFGIKLOPQ, and DHI-DFGKP strategy combinations. In addition, DHI-BGKR was also superior to the DHI-DFGKP strategy combination.

**Table 5:** Network meta-analysis of weight loss.

DHI^a^ strategy combinations	MDs^b^ (95% CIs)							
	DHI-BDFIQ^c^	DHI-BGKR^d^	DHI-ABCDEFGHIJKLNO^e^	DHI-BDFGIKLOPQ^f^	DHI-ABCDEFGHIJKLMNOQR^g^	DHI-EKP^h^	DHI-DFGKP^i^	Usual
DHI-BGKR	–3.50 (–6.35 to –0.65)	0	—^j^	—	—	—	—	—
DHI-ABCDEFGHIJKLNO	–4.10 (–7.46 to –0.74)	–0.60 (–3.55 to 2.35)	0	—	—	—	—	—
DHI-BDFGIKLOPQ	–5.00 (–7.71 to –2.29)	–1.50 (–3.69 to 0.69)	–0.90 (–3.72 to 1.92)	0	—	—	—	—
DHI-ABCDEFGHIJKLMNOQR	–5.70 (–12.66 to 1.26)	–2.20 (–8.97 to 4.57)	–1.60 (–8.60 to 5.40)	–0.70 (–7.41 to 6.01)	0	—	—	—
DHI-EKP	–5.90 (–12.00 to 0.20)	–2.40 (–8.28 to 3.48)	–1.80 (–7.94 to 4.34)	–0.90 (–6.72 to 4.92)	–0.20 (–8.85 to 8.45)	0	—	—
DHI-DFGKP	–6.24 (–8.58 to –3.91)	–2.74 (–4.43 to –1.05)	–2.14 (–4.59 to 0.31)	–1.24 (–2.69 to 0.20)	–0.54 (–7.11 to 6.03)	–0.34 (–5.99 to 5.30)	0	—
Usual	–6.50 (–8.82 to –4.18)	–3.00 (–4.66 to –1.34)	–2.40 (–4.83 to 0.03)	–1.50 (–2.92 to –0.08)	–0.80 (–7.36 to 5.76)	–0.60 (–6.24 to 5.04)	–0.26 (–0.54 to 0.03)	0

^a^DHI: digital health intervention.

^b^MD: mean difference.

^c^BDFIQ: communication, engagement, goal setting, management, and support.

^d^BGKR: communication, guide, monitor, and tailor.

^e^ABCDEFGHIJKLNO: action planning, communication, cues, engagement, feedback, goal setting, guide, identity, management, model or demonstrate, monitor, prompt, reward, and shape.

^f^BDFGIKLOPQ: communication, engagement, goal setting, guide, management, monitor, prompt, shape, stimulate, and support.

^g^ABCDEFGHIJKLMNOQR: action planning, communication, cues, engagement, feedback, goal setting, guide, identity, management, model or demonstrate, monitor, prompt, restructure, reward, shape, support, and tailor.

^h^EKP: feedback, monitor, and stimulate.

^i^DFGKP: engagement, goal setting, guide, monitor, and stimulate.

^j^Not applicable.

#### Heterogeneity and Consistency Analysis

The global inconsistency and local inconsistency were assessed using the node-splitting test for inconsistency analysis. The results showed no statistical inconsistency in each outcome comparison (*P*>.05). High heterogeneity between studies was only observed in BMI and weight loss outcomes. However, we did not find significant publication bias for these 2 outcomes through Egger test (*P*=.29 for BMI and *P*=.11 for weight loss). The net split function analyses found no statistically significant inconsistencies when assessing differences between direct and indirect effects. More details are provided in Table 6.

**Table 6:** The results of heterogeneity and inconsistency.

Outcome	Heterogeneity			Inconsistency (*P* value)
	*I*^2^ (%)	*P* value		
HbA_1c_^a^	7.3	.37	—^b^	
FBG^c^	0.0	.42	—	
BMI	95.8	<.001	—	
Weight loss	85.6	<.001	—	

^a^HbA_1c_: glycated hemoglobin A_1c_.

^b^Data not available.

^c^FBG: fasting blood glucose.

#### Assessment of Publication Bias

**Figure 6:**
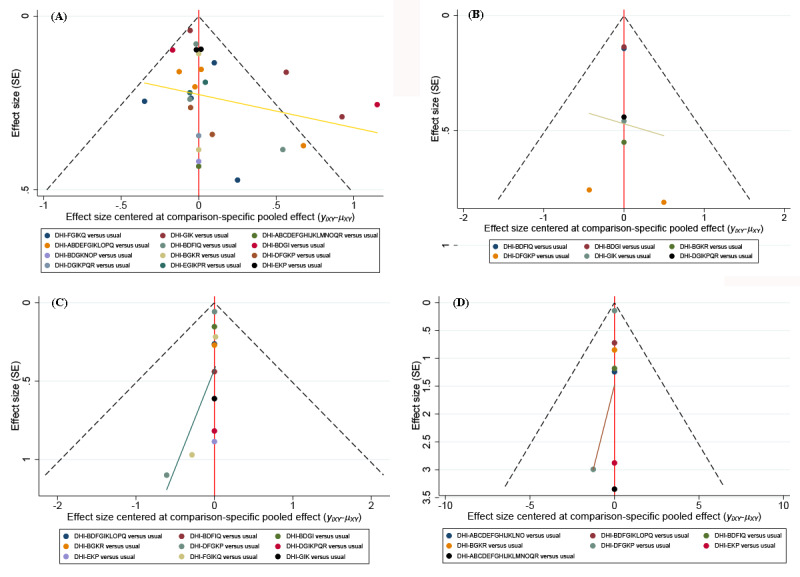
Comparison-adjusted funnel plots of trials included in the network meta-analysis: (A) comparison-adjusted funnel plots for glycated hemoglobin A1c, (B) comparison-adjusted funnel plots for fasting blood glucose, (C) comparison-adjusted funnel plots for BMI, and (D) comparison-adjusted funnel plots for weight loss. ABDEFGIKLOPQ: action planning, communication, engagement, feedback, goal setting, guide, management, monitor, prompt, shape, stimulate, and support; ABCDEFGHIJKLNO: action planning, communication, cues, engagement, feedback, goal setting, guide, identity, management, model or demonstrate, monitor, prompt, reward, and shape; ABCDEFGHIJKLMNOQR: action planning, communication, cues, engagement, feedback, goal setting, guide, identity, management, model or demonstrate, monitor, prompt, restructure, reward, shape, support, and tailor; BDFGIKLOPQ: communication, engagement, goal setting, guide, management, monitor, prompt, shape, stimulate, and support; BDFIQ: communication, engagement, goal setting, management, and support; BDGI: communication, engagement, guide, and management; BDGKNOP: communication, engagement, guide, monitor, reward, shape, and stimulate; BGKR: communication, guide, monitor, and tailor; DFGKP: engagement, goal setting, guide, monitor, and stimulate; DGIKPQR: engagement, guide, management, monitor, stimulate, support, and tailor; DHI: digital health intervention; EGIKPR: feedback, guide, management, monitor, stimulate, and tailor; EKP: feedback, monitor, and stimulate; FGIKQ: goal setting, guide, management, monitor, and support; GIK: guide, management, and monitor.

We conducted a comparison-adjusted funnel plot of trials included in the network meta-analysis for each result (Figure 6): (A) HbA_1c_, (B) FBG, (C) BMI, and (D) weight loss. The included studies were generally symmetrically distributed in the upper and middle parts of the funnel and around the left and right sides of the midline, indicating a low possibility of publication bias. Individual studies were distributed at the bottom, which may be related to the small sample size.

#### Sensitivity Analysis

**Figure 7:**
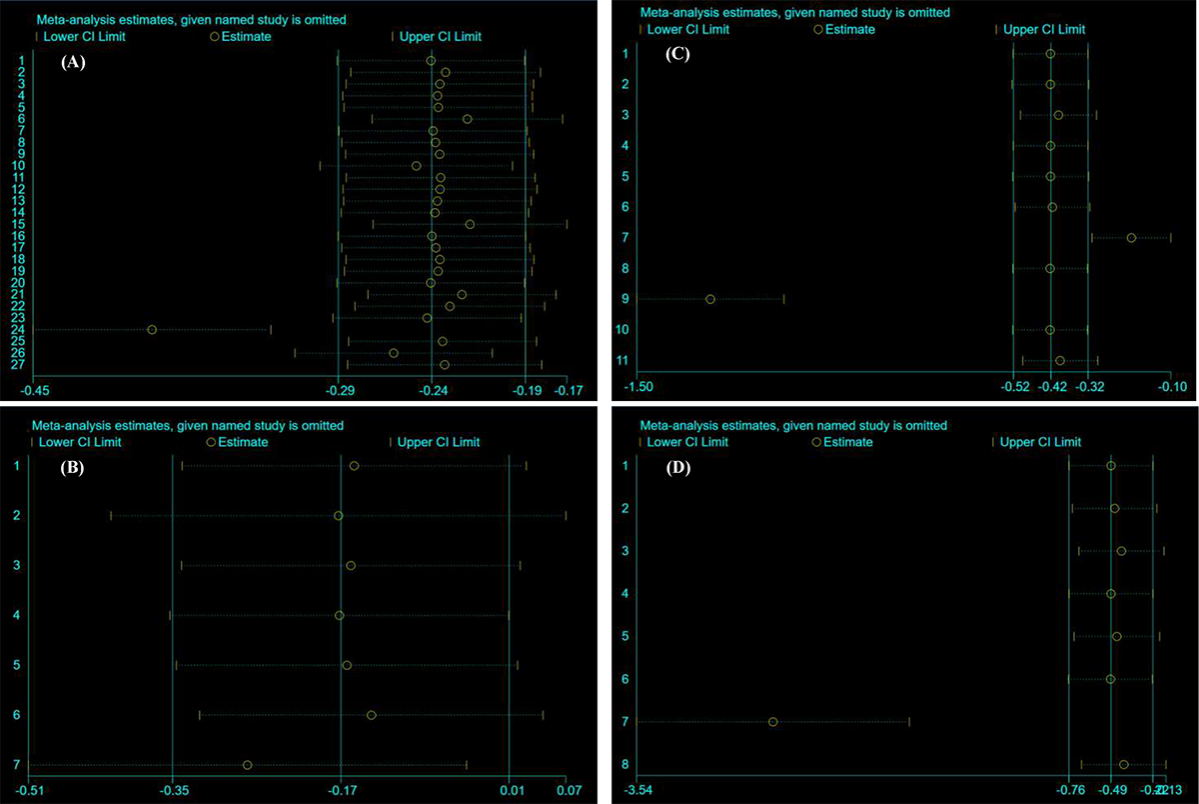
Sensitivity analyses using the method of removing individual studies separately: (A) sensitivity analyses for glycated hemoglobin A1c, (B) sensitivity analyses for fasting blood glucose, (C) sensitivity analyses for BMI, and (D) sensitivity analyses for weight loss.

We used the method of removing individual studies separately. Our results were generally stable and credible. As shown in Figure 7, the 2 vertical bright lines, except for the vertical axis, represent the CI of the merged population. Each study corresponds to 2 yellow short lines and a yellow circle, representing the CI of the remaining studies combined after excluding this study. If all yellow circles are within the range of 2 vertical bright lines, it indicates that this set of data is stable. Although the results showed that the combined effect value of HbA_1c_ levels, BMI, and weight loss appeared outside the CI of the original combined effect value, all CIs did not include 0; they were significant in treatment effects. The details are shown in Figure 7.

## Discussion

### Principal Findings

Our study offered a comprehensive insight into the strategies used in DHIs for T2DM management. A total of 52 RCTs were included, identifying 63 strategies categorized into 19 strategy themes. The most commonly used strategies were *guide*, *monitor*, *management*, and *engagement*. Most studies reported positive or mixed outcomes for most indicators based on the SF/HIT. Research involving a medium or high number of strategies was found to be more effective than research involving a low number of strategies. A total of 27 RCTs were included in the network meta-analysis. The strategy combination composed of *communication, engagement, guide*, and *management* was most effective in reducing HbA_1c_ levels, while the strategy combination that included *guide, management,* and *monitor* was effective in reducing FBG levels. A strategy combination composed of *communication, engagement*, *goal setting, management*, and *support* was most effective for BMI and weight management.

In the identified strategies, *monitor* and *guide* were the most frequently used ones, accounting for 77% (40/52) and 67% (35/52) of the total papers, respectively. Following closely were *management* (32/52, 61%) and *engagement* (28/52, 54%) strategies. The frequency of the use of these strategies indicates the trend and focus in this field. First, the *monitor* strategy reflects the role of digital tools in monitoring patients’ physiological indicators and behavior habits in diabetes management. It allows health care professionals and patients to track key parameters such as blood glucose level, weight, and exercise volume in real time, which helps to adjust the treatment plan in time and reduce the risk of complications [101]. Second, the use of a *guide* strategy reflects the value of digital health management in providing personalized and real-time advice and guidance to patients. Through intelligent algorithms and personalized settings, digital platforms can provide patients with accurate nutritional advice, exercise plans, and medication guidance based on their individual characteristics and historical data, helping them better manage their diseases and improve treatment outcomes [102]. These results are consistent with previous research that highlights the crucial role of *monitor* and *guide* strategies in DHIs [103,104]. On this basis, the *management* strategy emphasizes the important role of digital platforms in assisting health care professionals in disease management and treatment decision-making. Through data analysis and predictive models, digital platforms can provide health care professionals with more comprehensive and timely patient information, help them develop more effective treatment plans, and improve the accuracy and efficiency of clinical decision-making [105]. Furthermore, the *engagement* strategy emphasizes the promoting role of digital health management in patient participation and self-management. By providing personalized educational content, social support, and behavioral motivation, digital platforms can stimulate patients’ treatment motivation and enhance their awareness and self-management abilities toward the disease, thereby improving treatment compliance and long-term efficacy [106].

The number of strategies used in each study varies greatly, ranging from 1 to 32. Among 52 studies, 18 (35%) were categorized as high-strategy study, 23 (44%) as medium-strategy study, and 11 (21%) as low-strategy study. In digital health management, the use of a different number of strategies in research may reflect the diversity of research design, objectives, and methods. Many studies have chosen a medium number of strategies to comprehensively consider multiple aspects and evaluate the effectiveness of disease management. Research using a low number of strategies may focus more on the intervention effects in specific aspects to further explore the mechanisms of action of specific strategies. On the contrary, studies using a high number of strategies may aim to explore the combined effects of multiple intervention methods to achieve more comprehensive disease management. However, the results of the studies we included did not indicate that more strategies have more effective effects. Therefore, we further evaluated the impact of different numbers of strategies and compared the effect of different strategy combinations to provide more practical guidance for accurate and personalized digital health management.

We assessed the effectiveness of DHI strategies qualitatively based on the SF/HIT. Overall, most (≥50/52, ≥96%) studies have reported positive or mixed outcomes for most outcome indicators, such as preventive care; efficiency; perceived ease of use or usefulness; safety, privacy or security; acceptability; appropriateness; satisfaction process of service delivery or performance; effectiveness; and adherence or attendance. However, in terms of cost-effectiveness, most (50/52, 96%) studies have not reported positive results. This may be due to the introduction and development of digital health management tools, which provide more convenient, safe, and efficient management methods for patients with diabetes [60]. These tools include remote monitoring devices, mobile apps, and web-based platforms that can help patients better monitor blood glucose levels, manage diet, and engage in exercise, thereby improving the effectiveness of preventive care and service delivery processes and enhancing patient acceptance and satisfaction with treatment plans [105]. However, the negative results in terms of cost-effectiveness may be due to the significant investment required for the implementation and operation of digital health management tools, and the long-term cost-effectiveness has not been fully demonstrated [59,62,73]. In high-strategy and medium-strategy studies, most (36/41, 88%) studies reported positive outcomes, while low-strategy (7/11, 64%) studies reported fewer positive outcomes. This reflects the investment of more resources and technology, which can more comprehensively meet the needs of patients and improve treatment effectiveness. In contrast, low-strategy research may not have achieved the same effect due to resource constraints or insufficient technology. This was consistent with research by Brueton et al [35], which indicates that using a combination of strategies rather than a single approach can be more effective in enhancing participant retention. Future research should focus on cost-effectiveness analysis, explore the long-term economic benefits of digital health management tools, and propose more effective low-cost strategies to promote the sustainable development of diabetes management strategies.

In the meta-analysis, we included 27 studies evaluating the efficacy of 12 different DHI strategy combinations on HbA_1c_ levels. We found several high-quality combinations that can significantly reduce the HbA_1c_ levels, which reflects that, in diabetes management, using a specific strategy combination may have more advantages than using a single strategy. Among numerous strategy combinations, the SUCRA probability ranking suggested that DHI-BDGI (communication, engagement, guide, and management) might be the most effective strategy combination, followed by DHI-DFGKP (engagement, goal setting, guide, monitor, and stimulate); DHI-FGIKQ (goal setting, guide, management, monitor, and support); and DHI-ABDEFGIKLOPQ (action planning, communication, engagement, feedback, goal setting, guide, management, monitor, prompt, shape, stimulate, and support). The effect of only using the combination of DHI-GIK (guide, management, and monitor) was relatively weak. First, in the current medical practice, the treatment of diabetes is no longer a simple drug treatment but a comprehensive management, including lifestyle intervention, psychological support, drug treatment, and other comprehensive interventions [5]. Among these, communication and patient engagement are considered crucial factors that can enhance patients’ understanding and compliance with treatment and improve treatment effectiveness [106]. Meanwhile, guidance and management provide specific action guidelines and treatment plans to help patients better control their blood glucose levels [102,105]. The combination of these strategies complements each other and can comprehensively cover all aspects of the treatment process, maximizing the therapeutic effect. Second, these 2 combinations of DHI-DFGKP (engagement, goal setting, guide, monitor, and stimulate) and DHI-FGIKQ (goal setting, guide, management, monitor, and support) also showed good results. They emphasize aspects such as patient engagement, goal setting, guidance, monitoring, and support. The combination of these strategies makes the treatment process more systematic and orderly, helping patients better understand and execute treatment plans and improving the success rate of treatment [59]. However, the combination of DHI-ABDEFGIKLOPQ (action planning, communication, engagement, feedback, goal setting, guide, management, monitor, prompt, shape, stimulate, and support) encompasses more strategies and may seem more comprehensive, but there may be some challenges in practical applications. This complex combination may require more resources and time to implement while also increasing the cognitive burden on patients, which may affect treatment compliance. Finally, the combination of DHI-GIK (guide, management, and monitor), which only includes a few strategies, was relatively weak. This may be due to a lack of communication and patient engagement, reflecting insufficient communication between health care professionals and patients in real clinical practice and a lack of enthusiasm and participation from patients during treatment [78,106]. In addition, relying solely on guidance and management while ignoring the psychological and lifestyle factors of patients can easily lead to poor treatment outcomes.

At the same time, we included 7 studies evaluating the efficacy of 6 different DHI strategy combinations on FBG levels. Only DHI-GIK (guide, management, and monitor) was shown to have an effect. The advantage of this effective strategy combination is that it integrates 3 key elements: guidance, management, and monitoring. These strategies can provide systematic treatment support, help patients develop and execute effective treatment plans, and monitor treatment outcomes in a timely manner, thereby better controlling FBG levels [97]. Our result was consistent with research that has shown that multidimensional interventions, including guidance, management, and monitoring, are critical to the success of diabetes management [5]. However, other strategy combinations may lack sustained guidance and monitoring or may not fully motivate patients to actively participate in treatment, resulting in poor effect compared with DHI-GIK (guide, management, and monitor). Meanwhile, this result may also be influenced by the bias caused by the insufficient amount of original studies on FBG levels in our meta-analysis. Therefore, there is a certain difference in the results between studies evaluating FBG and HbA_1c_ levels.

In general, our research results highlight the importance of comprehensive strategy combination in blood glucose management of T2DM. Future research can further explore other combinations of strategies to find more effective management models for diabetes. There is a need to strengthen communication and interaction between health care professionals and patients, as well as to improve patient participation and compliance. Furthermore, it is essential to strengthen the long-term tracking and evaluation of diabetes management strategies.

This review showed that DHI-BDFIQ (communication, engagement, goal setting, management, and support) and DHI-BGKR (communication, guide, monitor, and tailor) combination strategies were significantly effective for both BMI and weight loss. Among these, DHI-BDFIQ (communication, engagement, goal setting, management, and support) was considered the optimal combination. First, it covers the following key aspects: effective communication promotes cooperation and understanding between patients and medical teams; active engagement enhances the patient’s participation and execution of treatment, such as physical activity and dietary intake control; clear goal setting helps guide the direction and progress of treatment [98]; effective management can provide systematic treatment plans and continuous management support; and continuous support can help patients overcome difficulties and maintain a positive attitude during the treatment process [59], to help patients better manage their weight. On the other hand, DHI-BGKR (communication, guide, monitor, and tailor) emphasizes personalized guidance and monitoring, which is more targeted, allowing patients to adjust their weight management plan according to their own situation [56,64,70], thereby reducing weight and BMI. In addition, the combination strategy of DHI-BDGI (communication, engagement, guide, and management) was found to be effective for BMI, and DHI-BDFGIKLOPQ (communication, engagement, goal setting, guide, management, monitor, prompt, shape, stimulate, and support) was effective for weight loss. Our research results have indicated that DHI-BDGI (communication, engagement, guide, and management) was the most effective combination strategy in reducing the HbA_1c_ levels, and therefore, it has a good effect on controlling BMI. Studies have shown that comprehensive treatment plans, including communication, engagement, guidance, and management, can effectively improve blood glucose control and weight management in patients with T2DM [107,108]. The effectiveness of these strategies may stem from their ability to comprehensively influence the patient’s lifestyle and behavioral habits, thereby promoting the achievement of treatment goals [73,79,94]. The combination of DHI-BDFGIKLOPQ (communication, engagement, goal setting, guide, management, monitor, prompt, shape, stimulate, and support) includes multiple strategies, from goal setting to behavior shaping and support. While it has a positive effect on weight loss, it may encounter some challenges in practical applications, leading to a relatively weak overall effect. Therefore, the effect was relatively weak.

Our research emphasizes the advantages of the combination of communication, patient engagement, goal setting, personalized management, external support, and continuous monitoring strategies in the DHIs of BMI and weight management of patients with T2DM. Future research can further explore the applicability of these strategy combinations in different outcomes, such as quality of life, complication rate, and other indicators. In addition, precise personalized intervention based on artificial intelligence and big data analysis is necessary. At the same time, more interactive and personalized digital health platforms can be developed to improve patient engagement and treatment compliance.

### Limitations

This study has several limitations. First, despite conducting a comprehensive search, it is possible that some relevant papers published in non-English languages or from nonindexed sources might have been overlooked. Second, we conducted 3 rounds of data screening and extraction, which could have introduced inconsistencies in certain cases. In addition, in our quantitative analysis, we only performed meta-analyses on specific outcome measures, thereby limiting the generalizability of our conclusions. Regarding BMI and weight loss outcomes, there was substantial heterogeneity among studies, which we addressed through sensitivity analyses and publication bias assessments. Furthermore, the number of original studies included in the meta-analyses was relatively small, potentially leading to some degree of bias in the conclusions. Finally, due to the nature of the interventions, blinding was not feasible in most studies. This might have exaggerated the estimated intervention effects in the network meta-analysis and contributed to the low to moderate quality of the evidence and the low methodology quality of included studies.

### Conclusions

Our research provided a comprehensive analysis and summary of the strategies used in DHIs for T2DM. We identified 63 strategies and categorized them into 19 strategy themes. *Guide*, *monitor*, *management*, and *engagement* were the most commonly used strategies. Most studies reported positive or mixed outcomes for most outcome indicators based on the SF/HIT. Research involving a medium or high number of strategies was found to be more effective than research involving a low number of strategies. The strategy combination composed of *communication*, *engagement*, *guide*, and *management* was most effective in reducing HbA_1c_ levels, while the strategy combination that included *guide*, *management*, and *monitor* was effective in reducing FBG levels. The strategy combination composed of *communication*, *engagement*, *goal setting*, *management*, and *support* was most effective in BMI and weight management. Future research should further confirm the effectiveness of these strategies in other indicators and populations, explore more strategy combinations, and optimize the design and implementation of strategies for patients with diabetes. Furthermore, it is necessary to develop more interactive and personalized digital health platforms. Finally, the cost-effectiveness analysis of strategy use should be strengthened to provide more effective guidance for disease management and clinical practice of T2DM.

## Appendix

Multimedia Appendix 1 PRISMA (Preferred Reporting Items for Systematic Reviews and Meta-Analyses) checklist.

Multimedia Appendix 2 Search strategy.

Multimedia Appendix 3 Behavior change techniques.

Multimedia Appendix 4 Characteristics of the included studies.

Multimedia Appendix 5 Detailed information about strategies and strategy combinations.

Multimedia Appendix 6 Evaluation of the effectiveness of different digital health intervention strategies based on the synthesis framework for the assessment of health IT.

Multimedia Appendix 7 Risk of bias and quality assessments of included studies.

## Abbreviations

DHI: digital health intervention
FBG: fasting blood glucose
HbA1c: glycated hemoglobin A1c
MD: mean difference
PRISMA-NMA: Preferred Reporting Items for Systematic Review and Meta-Analyses extension for Network Meta-Analysis
RCT: randomized controlled trial
SF/HIT: synthesis framework for the assessment of health IT
SUCRA: surface under the cumulative ranking curve
T2DM: type 2 diabetes mellitus
