# Supplementary material for: Assessing the Effectiveness of Digital Health Behavior Strategies on Type 2 Diabetes Management: Systematic Review and Network Meta-Analysis
Source: J Med Internet Res. 2025 Feb 14;27:e63209. doi: 10.2196/63209 (PMC11888087; doi:10.2196/63209)
Supplement: Multimedia Appendix 2 [file jmir_v27i1e63209_app2.docx]

Appendix 2. Search strategy (PubMed as an example)

| No. | PubMed Search strategy |
| --- | --- |
| #1 digital | “digital”[Title/Abstract] OR “wearable devices”[Title/Abstract] OR “telemedicine”[Title/Abstract] OR “electronic health records” OR “electronic medical records”[Title/Abstract] OR “mobile phone applications”[Title/Abstract] OR “web pages”[Title/Abstract] OR “blogs”[Title/Abstract] OR “emails”[Title/Abstract] OR “text messages”[Title/Abstract] OR “social media”[Title/Abstract] OR “emails”[Title/Abstract] OR “emails”[Title/Abstract] OR “technologies”[Title/Abstract] |
| #2 health-related | “health”[Title/Abstract] OR “health behavior improvement”[Title/Abstract] OR “disease treatment”[Title/Abstract] OR “health education” |
| #3 RCTs | “randomized controlled trial” [Title/Abstract] OR “randomized” [Title/Abstract] OR “placebo” [Title/Abstract] OR “clinical trial”[Title/Abstract] OR “RCT” [Title/Abstract] |
| #4 | 1 AND 2 AND 3 |
| #5 Time span | From 1 January 1999 to 10 March 2024 |
