# Supplementary material for: Assessing the Effectiveness of Digital Health Behavior Strategies on Type 2 Diabetes Management: Systematic Review and Network Meta-Analysis
Source: J Med Internet Res. 2025 Feb 14;27:e63209. doi: 10.2196/63209 (PMC11888087; doi:10.2196/63209)
Supplement: Multimedia Appendix 3 [file jmir_v27i1e63209_app3.docx]

**Behaviour Change Technique Taxonomy used in the sort tasks (for full BCTv1 Taxonomy see Michie, et al., 2013)**

| **BCT no.** | **Label** | **Definition** |
| --- | --- | --- |
|  | Antecedents | Record/provide information about antecedents  (*e.g., social and environmental situations and events, emotions, cognitions)* that reliably predict performance of the behaviour |
|  | Problem solving/coping planning | Analyse the problem and generate or select solutions that include overcoming barriers and increasing facilitators |
|  | Avoidance/changing exposure to cues for the behaviour | Advise on how to avoid exposure to specific social and contextual cues for the unwanted behaviour, including changing daily or weekly routines |
|  | Prompts/cues | Use environmental, social or internal stimuli to prompt or cue performance of wanted behaviour or non-performance of unwanted behaviour |
|  | Restructuring the physical environment | Change the physical environment in order to facilitate performance of the wanted behavior or create barriers to the unwanted behaviour (other than prompts, rewards and punishments) |
|  | Restructuring the social environment | Change the social environment in order to facilitate the wanted behavior or create barriers to the unwanted behaviour (other than prompts, rewards and punishments) |
|  | Fading | Withdraw prompts to perform the behaviour gradually |
|  | Habit reversal | Prompt rehearsal and repetition of an alternative behaviour to replace an unwanted habitual behaviour |
|  | Classical conditioning | Present a neutral stimulus jointly with a stimulus that already elicits a response repeatedly until the neutral stimulus elicits that response *(Pavlovian Conditioning)* |
|  | Escape learning | Arrange for the removal of an aversive stimulus |
|  | Discriminative (learned) cue | Identify an environmental stimulus that has been repeatedly associated with contingent reward for specified behaviour |
|  | Discrimination training | Reward the behaviour in one situation but not in another |
|  | Generalisation of a target behaviour | Once a behaviour is performed in a particular situation, encourage or help to try it in another situation |
|  | Pharmacological support | Provide or encourage the use or adherence to drugs to facilitate behaviour change *e.g., nicotine replacement, analgesics* |
|  | Social and environmental consequences | Record/provide information about objective social and environmental consequences of performing the behaviour |
|  | Health consequences | Record/provide information about health consequences of performing the behaviour |
|  | Emotional consequences | Record/provide information about emotional consequences of behaviour |
|  | Vicarious reinforcement | Facilitate observation of the consequences for others when they perform the target behaviour |
|  | Salience of consequences | Use methods to emphasise the consequences of changing the behaviour not only informing about consequences |
|  | Incentive | Inform that performance will be rewarded contingent on behaviour in the future |
|  | Self reward | Praise or reward if and only if there has been effort and/or progress made towards the behaviour |
|  | Social Reward | Verbally or non verbally reward (praise) if and only if there has been effort and/or progress made towards performing the behaviour |
|  | Material Reward | Provide money, vouchers or other valued objects if and only if there has been effort and/or progress made towards performing the behaviour |
|  | Shaping | Provide contingent rewards for any approximation to the target behaviour, gradually rewarding only performance closer to the wanted behaviour |
|  | Chaining | Build up behaviour by reinforcing final component; gradually add components earlier in sequence |
|  | Counter-conditioning | Reward for responding to a stimulus in a manner that is incompatible with a previous response to that stimulus |
|  | Thinning | Provide a gradual increase in the intermittency of reinforcement |
|  | Extinction | Discontinue contingent reward for performing the unwanted behaviour |
|  | Covert conditioning | Instruct to imagine performing a wanted behaviour in a real-life situation followed by imagining a pleasant consequence |
|  | Negative reinforcement | Arrange for removal of an unpleasant consequence contingent on the wanted behaviour |
|  | Punishment | Identify and provide aversive consequence contingent on the performance of the unwanted behaviour |
|  | Response cost | Withdraw something valued (not a contingent reward) as a consequence of performing an unwanted behaviour |
|  | Threat | Inform that future punishment or removal of reward will be contingent on performance of an unwanted behaviour (may include fear arousal) |
|  | Differential reinforcement | Arrange for reinforcement of alternatives to performing the unwanted behaviour |
|  | Time out | Separate person from situations in which behaviour can be reinforced in order to reduce an unwanted behaviour. |
|  | Satiation | Arrange repeated exposure to a stimulus that reduces or extinguishes a drive state |
|  | Covert sensitization | Instruct to imagine performing the unwanted behaviour in a real-life situation followed by imagining an unpleasant consequence |
|  | Overcorrection | When inappropriate behaviour is exhibited ask to repeat the behaviour in an appropriate but exaggerated way |
|  | Anticipated regret | Induce expectations of future regret about the performance of an unwanted behaviour or non-performance of a wanted behaviour |
|  | Anticipation of future rewards or removal of punishment | Inform that future rewards or removal of future punishment will be contingent on performance of wanted behaviour |
|  | Regulate negative emotions | Teach methods of reducing anxiety, stress and/or anger to facilitate performance of target behaviour |
|  | Exposure | Provide systematic confrontation with a feared stimulus |
|  | Behavioural contract | Create a written agreement on the performance of an explicitly specified behaviour witnessed by another |
|  | Goal setting (outcome) | Set a goal defined in terms of a positive outcome of wanted behaviour *(e.g., weight to help change eating patterns)*  *Note: If the goal is behavioural code Goal setting (behavioural)* |
|  | Review of outcome goal(s) | Review outcome goal(s) and modify goal in light of achievement |
|  | Goal setting (behaviour) | Set a goal defined in terms of the behaviour to be achieved |
|  | Review behaviour goal(s) | Review behaviour goal(s) and modify goal or behaviour change strategy in light of achievement |
|  | Action planning (including implementation intentions) | Prompt detailed planning of the behaviour goal (including at least one of context, frequency, intensity and duration of performance) |
|  | Discrepancy between current behaviour and goal standard | Draw attention to discrepancy (direction and amount) between standard and own behaviour (goes beyond simple self-monitoring). |
|  | Time management | Teach how to manage time in order to create opportunities when the wanted behaviour could be performed |
|  | Self-monitoring of behaviour | Instruct self recording of specified behaviour/s (with or without associated thoughts, emotions, situations) as part of a behaviour change strategy |
|  | Self-monitoring of outcome of behaviour | Establish self recording of outcomes of behaviour, *e.g., blood pressure, blood glucose, weight loss, physical fitness* |
|  | Other monitoring with awareness | Observe or record behaviour with the person’s knowledge |
|  | Feedback on behaviour | Provide feedback on behaviour *e.g., form, frequency, duration, intensity* |
|  | Biofeedback | Provide feedback about the body (e.g., *physiological or bio chemical state)* using an external monitoring device as a behaviour change strategy |
|  | Self assessment of affective consequences | After attempts at performing the behaviour, prompt assessment of feelings about performing the behaviour |
|  | Behavioural rehearsal /practice | Prompt rehearsal or practice of the performance of the behaviour one or more times. |
|  | Habit formation | Prompt rehearsal and repetition of the behaviour in the same context repeatedly |
|  | Instruction on how to perform a behaviour | Instruct, advise or agree the behaviour to be changed. |
|  | Conserving mental resources | Advise on ways of minimising demands on mental resources *e.g., getting enough sleep (cognitive), avoiding stressful situations (*emotional) |
|  | Self talk | Prompt positive self talk (aloud or silently) before and during the behaviour |
|  | Behavioural experiments | Identify and test hypotheses about the behaviour, its causes and consequences, by collecting and interpreting data |
|  | Modelling of the behaviour | Provide an example for people to aspire to or imitate |
|  | Paradoxical instructions | Instruct to engage in some form of the unwanted behaviour with the aim of reducing motivation to engage in the behaviour |
|  | Behaviour substitution | Facilitate substitution of the undesired behaviour |
|  | Tailored personalised message | Tailor the message, verbal or written, provided to the individual or group, based on their characteristics. |
|  | Graded tasks | Set easy-to-perform tasks, making them increasingly difficult until wanted behaviour is performed |
|  | Social support (general) | Advise on, facilitate or provide development of general social support for the behaviour *(e.g. friends, relatives, colleagues, ‘buddies’ or staff)* |
|  | Social support (emotional) | Advise on or facilitate development of emotional social support for performing the behaviour |
|  | Social support (practical) | Advise on or facilitate development of practical help for achieving the behaviour |
|  | Information about others’ approval | Provide information about what other people think about the behaviour. It clarifies whether others will like, approve or disapprove of what the person is doing or will do |
|  | Non-specific encouragement | Praise or reward for effort or performance without making this contingent on specific behavioural performance |
|  | Social comparison | Explicitly draw attention to others’ performance to elicit comparisons |
|  | Identity associated with changed behaviour | Facilitate the person to construct a new self-identity as someone who ‘used to engage with the unwanted behaviour’ |
|  | Pros and cons | Facilitate identification and comparison of reasons for wanting (pros) and not wanting to (cons) change the behaviour |
|  | Comparative imagining of future outcomes | Facilitate imagining and comparing future outcomes of changed versus unchanged behaviour |
|  | Identification of self as role model | Inform that one's own behaviour may be an example to others |
|  | Focus on past success | Instruct to think about or list previous successes in performing the behaviour (or parts of it) |
|  | Mental rehearsal of successful performance | Help to practice imagining performing the behaviour successfully in relevant contexts |
|  | Commitment | Elicit statements indicating strong commitment to change the target behaviour |
|  | Persuasive argument | Present verbal or visual arguments from a credible source in favour of or against the behaviour |
|  | Verbal persuasion to boost self efficacy | Tell the person that they can successfully perform the behaviour, arguing against self doubts and asserting that they can and will succeed |
|  | Distraction | Facilitate use of an alternative focus for attention to avoid triggers for unwanted behaviour |
|  | Cognitive dissonance | Create discomfort by drawing attention to discrepancies between current or past behaviour and self-image |
|  | Self-affirmation | Encourage writing or completing rating scales about a cherished value or personal strength as a means of affirming their identity in this domain |
|  | Reframing | Encourage the deliberate adoption of a different perspective on behaviour in order to change cognitions or emotions about performing the behaviour |
|  | Reattribution | Shape the perception of causes of behaviour *e.g., external or internal and stable or unstable* |
